# Arterial Stiffness Parameters Correlate with Estimated Cardiovascular Risk in Humans: A Clinical Study

**DOI:** 10.3390/ijerph16142547

**Published:** 2019-07-17

**Authors:** Małgorzata Tąpolska, Maciej Spałek, Urszula Szybowicz, Remigiusz Domin, Karolina Owsik, Katarzyna Sochacka, Damian Skrypnik, Paweł Bogdański, Maciej Owecki

**Affiliations:** 1 Department of Public Health, Poznan University of Medical Sciences, Rokietnicka St. 4, 60-806 Poznań, Poland; 2 Department of Treatment of Obesity, Metabolic Disorders and Clinical Dietetics, Poznan University of Medical Sciences, Szamarzewskiego St. 82/84, 60-569 Poznań, Poland

**Keywords:** arterial stiffness, cardiovascular risk, stiffness index, reflection index

## Abstract

Arterial stiffness is said to be a novel predictor of cardiovascular events. This study investigated the correlation between arterial stiffness parameters and the estimated cardiovascular disease risk (RISK) in a Polish cohort of patients divided by age, sex, and body-mass index (BMI). The cross-sectional study enrolled 295 patients who met the inclusion criteria. Subjects were divided into three age groups, four weight groups, and by gender. The stiffness of the vessels was assessed by the measurement of the stiffness index (SI) and reflection index (RI). An individual 10-year RISK was calculated for each patient using the Heart Risk Calculator algorithm by the American Heart Association. A correlation between the SI and estimated RISK was observed (r_S_ 0.42, *p* < 0.05). The strongest relationship was presented for women, the age group 40–54, and individuals with normal weight. The correlation between RI and calculated RISK was observed (r_S_ 0.19, *p* < 0.05), the highest correlation was noticed for people aged 40–54 and obese. In conclusion, both SI and RI are correlated with estimated cardiovascular risk, however SI seems to be more useful than RI to predict the individual risk of future cardiovascular events. Both of these can be measured using non-invasive techniques, which demonstrates their potential utility in clinical practice.

## 1. Introduction

Arterial stiffness is a laboratory factor reflecting the rigidity of the arterial wall. The resistance of the arterial wall to deformation depends on the structural and functional changes of arteries [1]. Recently, several studies have shown possible mechanisms that may contribute to increased arterial stiffness including atherosclerosis, vascular calcification, extracellular matrix degradation, inflammation, and aging [2,3,4].

Organs that are most frequently affected by arterial stiffness are the kidneys, brain, and heart. Decreased elasticity of arterial walls results in the use of greater power carrying increased pulse pressure, which damages blood vessels [5,6]. This leads to the increased performance of the heart, which can cause left ventricular hypertrophy and remodeling [7]. Arterial stiffness is followed by elevated afterload and impaired coronary blood flow [8]. High levels of arterial stiffness may be the reason for increased levels of systolic (SBP) and diastolic blood pressure (DBP), which may be associated with increased morbidity and mortality for cardiovascular events [9,10].

Regardless of the pathophysiological paths, arterial stiffness is said to be a novel predictor of cardiovascular events, which are the leading causes of death worldwide [9,11,12]. Cardiovascular diseases are responsible for almost 19 million deaths per year in 2015, and it is estimated that this number will increase to more than 23.6 million by 2030 [13].

Selecting a reliable tool to estimate cardiovascular risk is a difficult task due to a wide range of potential factors. One of these tools is the Heart Risk Calculator, designed by the American College of Cardiology/American Heart Association (ACC/AHA), which assesses an individual’s 10-year risk of heart disease or stroke. The spreadsheet comprises age (from 40 to 79 years), gender, race (African American or other), total cholesterol, HDL cholesterol, SBP, the presence of hypertension therapy, diabetes, and smoking [14]. The ACC/AHA calculator is a combination of the known risk factors, the importance of which is commonly admitted. Nevertheless, traditional factors do not fully illustrate the real cardiovascular risk, and for this reason, novel, precise risk factors are under research including arterial stiffness [15,16,17].

In this paper, we investigated the association between arterial stiffness and cardiovascular risk based on the ACC/AHA calculator. The objective was to estimate whether there was a correlation between cardiovascular risk assessed by the Heart Risk Calculator and arterial stiffness parameters and, if present, whether this correlation differed by sex, age, and body mass index (BMI).

## 2. Materials and Methods

The study was performed on a group of 295 patients from the University Hospital of Lord’s Transfiguration (Poznań, Poland). The study was conducted from 2015 to 2017. The study was approved by the Ethical Committee of the Poznan University of Medical Sciences (approval number 359/15). Written informed consent was obtained from all participants.

The inclusion criteria for the study were aged from 40 to 79 years, total cholesterol concentration in blood serum from 130 to 320 mg/dL, HDL concentration in blood serum from 20 to 100 mg/dL, and SBP values from 90 to 200 mmHg. Except for hypertension, hyperlipidemia, diabetes, and obesity, the study participants did not suffer from other disorders and were otherwise healthy and in good clinical condition. Specifically, the exclusion criteria were a history of myocardial infarction, stroke, percutaneous coronary intervention, coronary artery bypass surgery, atrial fibrillation and other arrhythmias, and kidney, heart, or liver failure.

Each patient was evaluated regarding the total cholesterol and HDL cholesterol concentrations in the blood serum, measurements of body weight, height, SBP, measurements of the stiffness index (SI), and the reflection index (RI). Each patient filled a questionnaire in which information about suffering from diabetes, smoking, or taking antihypertensive drugs was collected.

### 2.1. Total Cholesterol, LDL- and HDL-Cholesterol Levels Measurements

The serum concentration of total cholesterol and HDL-cholesterol was measured in a commercial laboratory with the use of commercial kits. The serum concentration of LDL-cholesterol was calculated with Friedewald’s formula.

### 2.2. Body Mass and Height Measurements

The measurements were taken in the morning, 12 h from the last meal. Measurements were made as follows: body mass using the certified electronic weighing scale of the company Radwag (Radom, Poland) with a measuring accuracy of 0.1 kg; and height in an upright standing position without shoes, with an accuracy of 0.5 cm by using a measuring rod, which is an integral part of the weighing scale. On the basis of the performed measurements, BMI, defined as the body mass divided by the square of the body height, was calculated for each patient [18].

### 2.3. Blood Pressure Measurements

The traditional measurement of arterial blood pressure was carried out using a manual sphygmomanometer (Digital electronic tensiometer (model 705IT, Omron Corporation™, Kyoto, Japan)), following the European Society of Hypertension and European Society of Cardiology (ESH/ESC) recommendations from 2013 [19]. Blood pressure measurements were taken first on both arms. In the case of a pressure difference >10 mmHg, the higher value of measurement was registered. Measurement was carried out twice, the second time after 3–5 min rest, and in the case where the values were significantly different, the measure was averaged out. During the measurement, the patient sat in a chair with his arm resting on the table so that the elbow flexion was at the level of the heart. During the measurement, the lower edge of the cuff was 2–3 cm above the elbow flexion. After examining the pulse on the radial artery, the cuff was inflated to a value of 30 mmHg, above which the pulse on the radial artery was lost. Air from the cuff was released at 2 mmHg/s. Both pressure values were measured with an accuracy of 2 mmHg.

### 2.4. Assessment of Arterial Stiffness

The Pulse Trace PCA 2 apparatus (Micro Medical, Rochester, UK) was used to assess the stiffness of the vessels. This apparatus is used for non-invasive assessment of the structure and function of vessels. The European Network for Non-invasive Investigation of Large Arteries lists this apparatus among the reference methods for assessing vascular stiffness [20]. PCA 2 assesses the SI obtained by photopletysmography using a reader on the index finger. The measurement allows for the reconstruction of the pulse wave curve. The volume of the pulse wave (DVP; digital volume pulse) consists of two components: a systolic (the primary wave), which is the result of transmitting the pressure from the aorta to the arteries of the finger, and diastolic component (the second part of the wave), arising as a result of the return flow toward the aorta. The SI is defined as the quotient of the patient’s height and the time of reflection of the PPT wave (PPT; peak-to-peak time). That is, the difference between the first and the second peak of the pulse wave. SI is used to assess the stiffness of large vessels. RI describes the voltage of small vessels [21] and is calculated as the ratio between the amplitudes of the second and first peaks of the DVP waveform. It has been shown that this parameter can be used to assess the function of the vascular endothelium [22]. The examination of all patients was performed in an office with a temperature around 21 °C. The sensor was placed on the index finger of the hand in which the higher blood pressure was found. The test was performed in a lying position. The result was a mean of three measurements carried out each time between 30–45 s for each patient. In the absence of a typical waveform shape or large fluctuations of the SI index (>15%), the device reported the need to repeat the measurement. Patients were asked not to consume alcohol and coffee a day before the measurement and at the day of measurement. It should be emphasized that the results of this study might be unreliable in patients with arrhythmias, so for this reason, such patients were not eligible for the project.

### 2.5. Assessment of Cardiovascular Risk

An individual 10-year cardiovascular risk was calculated for each patient using the Heart Risk Calculator algorithm developed by the ACC/AHA. The calculator takes into account age, sex, race, SBP, total cholesterol, HDL cholesterol, the presence of diabetes, smoking, and the use of antihypertensive drugs. Calculated cardiovascular risk means the estimated 10-year risk of a first hard atherosclerotic cardiovascular event [14].

### 2.6. Statistics

All calculations were performed using the Statistica 13.1 program package from Statsoft. The normality of the distribution was checked by the Kolmogorov–Smirnov test. A significance level of 0.05 was assumed. The relationship between SI and RISK (%) was checked. Rank order correlations were calculated using Spearman’s rank order. The strength of the correlation relationship based on the linear correlation coefficient is as follows:

less than 0.2 = weak correlation (virtually no relationship)

0.2–0.4 = low correlation (explicit relationship)

0.4–0.6 = moderate correlation (significant relation)

0.6–0.8 = high correlation (significant dependence)

0.8–0.9 = very high correlation (very high dependence)

0.9–1.0 = the relationship is practically full

Patients were divided according to age (groups 40–54, 55–64, 65 or older), gender, and BMI (underweight = 16–18.5 kg/m^2^, normal weight = 18.5–24.99 kg/m^2^, overweight = 25–29.99 kg/m^2^, obesity >30 kg/m^2^). The number of patients in the age categories were as follows: 85 people in the 40–54 group, 121 in the 55–64 group, and 89 patients in the 65 or older group. The number of women and men was 204 and 91, respectively. A total of 126 obese patients, 107 overweight patients, 61 patients with normal weight, and one underweight person were included.

The second analysis concerned RI and RISK (%). The same program, the same level of significance, and strength of correlation were assumed. Patients were divided as above.

Additionally, relationships between SI and each of the following factors: age, BMI, SBP, DBP, total cholesterol, HDL cholesterol, and LDL cholesterol were checked using Spearman’s rank order. Similarly, the relationships between RI and each of the mentioned parameters were calculated.

## 3. Results

A total of 295 subjects were examined in the study where 69.15% were women (average age 59.02 (9.24) years old) and 41% of the subjects were between 54 and 65 years of age. From all patients, 17.63% were smokers, 9.83% had diabetes mellitus, and 44.07% were treated for hypertension. A total of 126 individuals were obese, and 107 were overweight. Data regarding the studied group are reported in Table 1.

A significant correlation between SI and RISK was found for all age categories, both genders, and all weight groups, where it was the strongest in the normal weight group (see Table 2). Significant correlation between RI and RISK was found for the age ranges 40–54 and 55–64, but not in the 65 years or older patients. From the BMI categories, a significant correlation was found only for obesity (see Table 3).

The results of the correlations between the stiffness parameters and cardiovascular risk factors above-mentioned are presented in Table 4.

## 4. Discussion

Arterial stiffness is increasingly considered as a marker of cardiovascular disease, and many studies have indicated its predictive role for cardiovascular diseases [9,23,24,25,26,27]. Data on arterial stiffness have demonstrated the relationship between stiffness parameters and many well-known risk factors such as hypercholesterolemia, diabetes, smoking, or hypertension [28,29,30,31]. In this study, we assumed that a mathematical algorithm comprising many features illustrated the cardiovascular risk more precisely than any of these factors separately.

Said et al. reported that stiffness index (SI) was a predictor of cardiovascular disease and mortality on the group of 169,613 subjects from the UK Biobank after a median follow-up of 2.8 years [32]. In a study by Ashan Gunarathne, an association between stiffness index and cardiovascular risk was investigated using the ESC HeartScore risk calculator in a cohort of 247 individuals [33]. In contrast to our study, in the Gunarathne survey, individuals with abnormal blood pressure measurements, fasting blood sugar level, or a total cholesterol level were excluded from the study. In agreement with our study, an association between SI and cardiovascular risk was presented [33]. In our study, we observed that SI also correlated with systolic blood pressure, diastolic blood pressure, total cholesterol level, and age, but the coefficients of these correlations were lower than for the relationship between SI and RISK. SI, therefore, most probably reflects the effect of many factors and better describes the risk than each of these factors separately.

Moreover, we observed a stronger correlation between stiffness index and RISK for females than for males. The strength of correlation also depended on the age group; the younger the group of patients, the stronger the dependence. The strongest correlation between risk and SI was also observed for normal weight individuals. The stronger dependence between the risk of cardiovascular disease and stiffness of vessels in younger and normal weight patients makes it possible to use this marker as an early indicator when the classic risk factors do not indicate the need for intervention. This gives the potential possibilities for the implementation of appropriate prophylaxis to prevent the occurrence of cardiovascular events in the future.

Furthermore, the influence of obesity on the usefulness of stiffness markers also remains unclear. Our study showed that while SI seems less useful in the group with excess body weight, RI, and perhaps other indicators of vessels stiffness may be more useful in the group of patients with obesity. It is worth mentioning that obesity itself causes increased cardiac output and can affect the stiffness measurements of the vessels. Conflicting results investigating the relationship between obesity and stiffness have been published thus far [34,35,36,37], which means that this issue requires further research.

Our study also investigated the second parameter of arterial stiffness, which is the reflection index, which opens up the possibilities to compare the usefulness of the stiffness index and reflection index. There are insufficient data about the usefulness of the reflection index; nevertheless, several studies have shown that the reflection index is a predictor of future cardiovascular events [38]. The ASCOT study by Manisty et al. showed that the reflection index is a predictor of a cardiovascular event in a 6.8-year follow-up of well-controlled hypertensive individuals independent of other risk factors [38]. Wang et al. investigated the value of arterial wave reflection on the group of 1272 normotensive and untreated hypertensive individuals from Taiwan and demonstrated that RI was predictive for all-cause and cardiovascular mortality, but only for men [39]. In the current study, we demonstrated the correlation between RI and estimated cardiovascular risk, although the Spearman’s correlation coefficient was lower here than the correlation between SI and estimated risk (0.19 compared to 0.42). Among the relationships between RI and the following factors of age, total cholesterol, HDL cholesterol, LDL cholesterol, SBP, DBP and BMI, only age and DBP were observed as factors correlating with the reflection index. The reflection index also correlated with RISK in the group of 40–54 aged patients.

Contrary to SI, the strongest correlation was observed in the group of obese individuals, which can be explained by the fact that RI reflects the dysfunction of endothelium, which can be impaired in obesity [40]. It creates a possibility of using RI in the detection of endothelial defects. The meta-analysis by Cote showed a correlation between obese children and adolescents and arterial stiffness [41], what can widen the usefulness of the measurement of RI. 

Some limitations of the study should be noted. First, the measurements performed by Pulse Trace device were considered to have suboptimal reproducibility [21]. The limitations of this research also result from the cross-sectional character of the study. Based on this study, it cannot be concluded that the analyzed parameters of arterial stiffness are predictive for cardiovascular events as the patients were not observed in follow-up studies. However, the strength of our study is that we used non-invasive methods to measure arterial stiffness, which enables us to advocate for the broader use of this tool in daily clinical practice. Importantly, SI and RI measurements may be of great value in younger people, where the assessment of cardiovascular risk with the use of traditional score sheets frequently faces difficulties.

## 5. Conclusions

In conclusion, SI and RI correlated with an individual 10-year cardiovascular risk measured by the AHA calculator mathematical algorithm, therefore they can be useful in the detection of patients who are at risk of future cardiovascular events. Additionally, both of these can be measured using non-invasive techniques, which demonstrates their potential utility in daily clinical practice. The highest usefulness of the stiffness index seems to be in the younger group of patients between 40 and 54 years old with the correct body mass. The reflection index seems to be less useful than SI, nevertheless it may be considered as an additional measurement, especially for subjects aged between 40 and 54 years old, or with obesity.

## Figures and Tables

**Table 1:** Group characteristics. F = female, M = male, SD = standard deviation, BMI = body mass index, SBP = systolic blood pressure, DBP = diastolic blood pressure, SI = stiffness index, RI = reflection index, RISK = cardiovascular risk based on ACC/AHA Heart Risk Calculator.

Feature	Value
Gender [F (%)/M (%)]	204 (69.15%)/91 (30.85%)
Age (Mean ± SD) (years)	59.02 ± 9.24
BMI (Mean ± SD) (kg/m^2^)	28.96 ± 5.07
SBP (Mean ± SD) (mmHg)	139.9 ± 19.5
DBP (Mean ± SD) (mmHg)	83.2 ± 10.7
Total cholesterol (Mean ± SD) (mg/dL)	206.7 ± 36.6
HDL cholesterol (Mean ± SD) (mg/dL)	63.4 ± 14.9
LDL cholesterol (Mean ± SD) (mg/dL)	112.4 ± 35.5
SI (Mean ± SD) (m/s)	7.86 ± 2.28
RI (Mean ± SD) (%)	54.51 ± 14.14
RISK (Mean ± SD) (%)	10.14 ± 10.97

**Table 2:** Spearman’s correlation between the stiffness index and cardiovascular risk based Heart Risk Calculator in groups divided by gender, age and body mass index. BMI = body mass index; *p*-value < 0.05 is indicated in bold.

	Number of Patients (%)	r_S_-Value	*p*-Value
All Patients	295 (100)	0.42	**<0.001**
***Gender***			
Female	204 (69.15)	0.39	**<0.001**
Male	91 (30.85)	0.36	**<0.001**
***Age***			
40–54	85 (28.81)	0.38	**<0.001**
55–64	121 (41.02)	0.30	**<0.001**
65 or older	89 (30.17)	0.29	**0.005**
***BMI***			
Underweight	1 (0.34)		
Normal weight	61 (20.68)	0.50	**<0.001**
overweight	107 (36.27)	0.30	**0.002**
Obesity	126 (42.71)	0.45	**<0.001**

**Table 3:** Spearman’s correlation between reflection index and cardiovascular risk based on Heart Risk Calculator in groups divided by gender, age and body mass index. BMI = body mass index; *p*-value < 0.05 is indicated in bold.

	Number of Patients (%)	r_S_-Value	*p*-Value
All patients	295 (100)	0.19	**<0.001**
***Gender***			
Female	204 (69.15)	0.12	0.091
Male	91 (30.85)	0.03	0.748
***Age***			
40–54	85 (28.81)	0.24	**0.026**
55–64	121 (41.02)	0.18	**0.049**
65 or older	89 (30.17)	0.16	0.124
***BMI***			
Underweight	1 (0.34)		
Normal weight	61 (20.68)	0.09	0.493
Overweight	107 (36.27)	0.12	0.210
Obesity	126 (42.71)	0.24	**0.007**

**Table 4:** Spearman’s correlation between arterial stiffness parameters and cardiovascular risk indicators. BMI = body mass index, SBP = systolic blood pressure, DBP = diastolic blood pressure; *p*-value < 0.05 is indicated in bold.

Indicator	Stiffness Index (SI)		Reflection Index (RI)	
	r_S_-Value	*p*-Value	r_S_-Value	*p*-Value
Age	0.34	**<0.001**	0.12	**0.041**
BMI	0.06	0.333	0.02	0.711
SBP	0.20	**<0.001**	0.07	0.215
DBP	0.25	**<0.001**	0.18	**0.002**
Total cholesterol	0.12	**0.044**	0.02	0.755
HDL cholesterol	−0.09	0.127	−0.08	0.163
LDL cholesterol	0.09	0.139	0.00	0.998
